# *STAT3* Genotypic Variant *rs744166* and Increased Tyrosine Phosphorylation of STAT3 in IL-23 Responsive Innate Lymphoid Cells during Pathogenesis of Crohn's Disease

**DOI:** 10.1155/2019/9406146

**Published:** 2019-06-19

**Authors:** Ying Tang, Sanda A. Tan, Atif Iqbal, Jian Li, Sarah C. Glover

**Affiliations:** ^**1**^ Department of Medicine, University of Florida, Gainesville, FL 32610, USA; ^2^Department of Surgery, University of Florida, Gainesville, FL 32610, USA; ^3^Department of Surgery, Baylor College of Medicine, Houston, TX 77030, USA; ^4^Department of Neurology, Thomas Jefferson University, Philadelphia, PA 19107, USA

## Abstract

Crohn's disease (CD) results from dysregulated immune responses to gut microbiota in genetically susceptible individuals, affecting multiple areas of the gastrointestinal tract. Innate lymphoid cells (ILCs) are tissue-resident innate effector lymphocytes which play crucial roles in mucosal immune defense, tissue repair, and maintenance of homeostasis. The accumulation of IFN-*γ*-producing ILC1s and increased level of proinflammatory cytokines produced by ILCs has been observed in the inflamed terminal ileum of CD patients. To date, the precise mechanisms of ILC plasticity and gene regulatory pathways in ILCs remain unclear. Signal transducer and activator of transcription 3 (STAT3) regulates gene expression in a cell-specific, cytokine-dependent manner, involving multiple immune responses. This study proposes the positive correlation between the prevalence of *STAT3 rs744166* risky allele “A” with the severity of disease in a cohort of 94 CD patients. In addition, the results suggest an increased STAT3 activity in the inflamed ileum of CD patients, compared to unaffected ileum sections. Notably, IL-23 triggers the differentiation of CD117^+^NKp44^−^ ILC3s and induces the activation of STAT3 in both CD117^+^NKp44^−^ and CD117^−^NKp44^−^ ILC subsets, implying the involvement of STAT3 in the initiation of ILC plasticity. Moreover, carriage of *STAT3* “A” risk allele exhibited a higher basal level of STAT3 tyrosine phosphorylation, and an increased IL-23 triggered the pSTAT3 level. We also demonstrated that there was no delayed dephosphorylation of STAT3 in ILCs of both A/A and G/G donors. Overall, the results of this study suggest that IL-23-induced activation of STAT3 in the CD117^−^NKp44^−^ ILC1s involves in ILC1-to-ILC3 plasticity and a potential regulatory role of ILC1 function. Those genetically susceptible individuals carried *STAT3 rs744166* risky allele appear to have higher basal and cytokine-stimulated activation of STAT3 signal, leading to prolonged inflammation and chronic relapse.

## 1. Introduction

Crohn's disease (CD) is one type of inflammatory bowel disease (IBD) that involves a chronic relapsing inflammation of the gastrointestinal tract, causing abdominal pain and diarrhea and can eventually lead to severe strictures or fistulae which enhance the need for surgical intervention [1]. The pathogenesis of CD involves a complex interaction between genetic factors, environmental factors, and dysregulated immune responses [2]. To date, the precise mechanisms behind the disease initiation and progression remain unclear. However, it is widely accepted that CD involves an inappropriate and continuing mucosal inflammatory immune response to intestinal microflora in genetically susceptible individuals [3, 4]. Historically, the dysfunctional adaptive immune responses were investigated in terms of Th1/Th17 responses in CD patients, and results suggested that T cells are responsible for the increased production of proinflammatory cytokines, such as IFN-*γ* and IL-17A [5]. Recently, increasing evidence suggests that innate immunity also plays a significant role in the pathogenesis of CD [6, 7]. Innate lymphoid cells (ILCs) have been identified as innate effector cells that are involved in bacterial defense, induction of inflammation, and the maintenance of homeostasis [8]. The nomenclature of ILCs is based on their signature cytokines and the transcriptional factors that regulate their differentiation. They are classified into natural killer (NK) cells, group 1 ILCs (ILC1s), group 2 ILCs (ILC2s), group 3 ILCs (ILC3s), and lymphoid tissue inducer (LTi) cells [9]. In contrast to T cells, ILCs lack antigen-specific receptors and are constitutively located in barrier tissues, responding to alarmins as well as cytokine signals released following tissue damage or mucosal infection [10, 11]. As tissue-resident innate compartments of T helper cells, ILCs undergo transdifferentiation, also known as plasticity, in response to cytokine cues in the surrounding tissue microenvironment [12]. Specifically, in Crohn's disease patients, we and other researchers have identified the accumulation of IFN-*γ*-producing ILC1s at the expense of IL-17/IL-22-producing ILC3s in the inflamed intestinal tissues of Crohn's disease patients, implying this ILC3-to-ILC1 plasticity may be involved in the CD pathogenesis [13, 14]. Moreover, researchers have identified that the differentiation toward ILC1s was driven by IL-12, and conversely, IL-23 promoted polarization toward ILC3s *in vitro* and *in vivo* [15]. Notably, IL-23 responsive ILCs have been recently recognized as the important cell population that associates with the pathogenesis of colitis in several murine models [16].

Genome-wide association studies (GWAS) have identified 170 disease susceptibility gene loci that are associated with Crohn's disease [17]. In 2014, a meta-analysis indicated that the *STAT3 rs744166* polymorphism was highly associated with CD susceptibility, especially among Caucasians [18]. Additionally, the *STAT3 rs744166* risk allele “A” has been associated with increased cellular STAT3 activation in leukocytes in pediatric Crohn's disease patients [19]. Signal transducer and activator of transcription 3 (STAT3) is a crucial transcription factor which regulates cells associated with both innate and adaptive mucosal immunity in a cell-specific, cytokine-dependent manner [20]. Particularly, STAT3 plays a central role in Th17 commitment through its direct regulation of ROR*γ*t and regulates Th17-cytokine production in response to IL-23 [21]. In addition, it has been shown that STAT3 is essential for ILC3s to produce IL-22 against intestinal infection in mice [22, 23]. Recent improvements in genetic analysis in human studies have revealed the distinct gene regulatory mechanisms in adaptive and innate lymphoid cells [24, 25]. Although ILC3s are the innate analog of Th17 cells, the exact regulation of STAT3 in ILC3 development and plasticity remains unclear. In this study, we investigate the role of STAT3 in the pathogenesis and progression of Crohn's disease, and more specifically, we examine the STAT3 signaling involving in innate lymphoid cell plasticity.

## 2. Materials and Methods

### 2.1. Human Intestinal Lamina Propria Mononuclear Cell (LPMC) Isolation

All the surgical resections of the terminal ileum from Crohn's disease patients were obtained from UF Health Shands Hospital, following an approved IRB#201500440. LPMCs were isolated by epithelial elimination and enzyme digestion. Briefly, the surgical resections were cut into 1-2 mm pieces after removal of the outer fat tissue and muscle layer. The epithelial layer was removed by 1X Hanks' Balanced Salt Solution (HBSS) with phenol red and glucose, no calcium, and no magnesium, containing 2% FBS (Gibco®), 100 U/ml penicillin, 100 *μ*g/ml streptomycin, 25 *μ*g/ml gentamycin, and 0.5 mM DTT. Tissues were then digested with 1X HBSS containing 1% FBS (Gibco®), 100 U/ml collagenase XI (Roche), 20 *μ*g/ml Dispase neutral protease II (Roche), and 10 *μ*g/ml deoxyribonuclease I (StemCell Technologies). The remaining tissues were mechanically dissociated and were passed through 70 *μ*m cell strainers (Corning®). All cells were then slowly cryopreserved in CryoStor® CS10 (StemCell Technologies) and transferred into liquid nitrogen for future use.

### 2.2. Human Peripheral Blood Mononuclear Cell (PBMC) Isolation

Donor blood was purchased from LifeSouth Community Blood Center, and patient blood was obtained from UF Health Shands Hospital, following an approved IRB#201500440. PBMCs were isolated by gradient centrifugation. Briefly, whole blood samples were diluted 1 : 1 ratio with 1XPBS containing 2% FBS. Diluted blood samples were carefully transferred into SepMate™-50 tubes containing 15 ml of Lymphoprep™ (StemCell Technologies). Samples were then centrifuged at 1,200xg for 10 mins, and the cell suspensions above the insert in SepMate™-50 tubes were transferred into new 50 ml conical tubes. PBMCs were collected by centrifuging at 300xg for 8 mins. All cells were then slowly cryopreserved in CryoStor® CS10 (StemCell Technologies) and transferred into liquid nitrogen for future use.

### 2.3. Genotyping of *STAT3 rs744166*

Genomic DNA was extracted from whole blood using the Gentra Puregene Kit (QIAGEN). Patients were genotyped for the *STAT3 rs744166* (assay ID: C___3140282_10, Cat. No. 4351379, TaqMan) single nucleotide polymorphism (SNP) using the TaqMan system, running on the QuantStudio 7 Flex Real-Time PCR System (Applied Biosystems).

### 2.4. Quantitative RT-PCR

Total RNA was isolated from intestinal tissues of Crohn's disease patients using QIAzol Lysis Reagent (QIAGEN) or from whole blood of CD patients using the RNeasy Kit (QIAGEN). A two-step RT-PCR was performed using the SuperScript® VILO cDNA Synthesis Kit (Thermo Fisher Scientific) for reverse transcription (RT), and PCR was done using the QuantStudio 7 Flex Real-Time PCR System (Applied Biosystems). The PCR was performed using a 20 *μ*l reaction volume containing the cDNA, 2× TaqMan® Gene Expression Master Mix, nuclease-free water, and 1× TaqMan Gene Expression Assay for STAT3 (Thermo Fisher Scientific). The housekeeping gene GAPDH was used as an endogenous control to normalize target gene expression levels. 2^-*ΔΔ*Ct value was calculated and used to represent the gene expression fold change.

### 2.5. Immunohistochemistry

The immunohistochemistry staining followed the manufacturer's protocols, and the heat-induced epitope retrieval method with citrate buffer was used for antigen retrieval. Primary antibodies, specifically anti-STAT3 (Y705) antibody (rabbit polyclonal, ab214465 (dilution 1:500), Abcam), were added and incubated at 4°C overnight. The primary antibodies were detected using a mouse and rabbit specific HRP/DAB IHC detection kit (ab236466, Abcam) following the manufacturer's instructions.

### 2.6. Flow Cytometry

Single-cell suspensions isolated from intestinal tissues of CD patients were stained with FITC-conjugated anti-human lineage cocktail 3 (Lin3) (CD3, CD14, CD19, and CD20; clone: M*φ*P9, L27, SK7, and SJ25C1; BD Biosciences), PerCP-conjugated anti-human CD45 (clone: HI30, BioLegend), APC-conjugated anti-human CD127 (clone: A019D5, BioLegend), PE-conjugated anti-human NKp44 (clone: P44-8, BioLegend), PE-Cy7-conjugated anti-human CD117 (clone: 104D2, eBioscience), Brilliant Violet 421-conjugated anti-human CRTH2 (clone: BM16, BioLegend), and Alexa Fluor 647-conjugated anti-STAT3 Phospho-Tyr705 (clone: 13A3-1, BioLegend). Viability was assessed by Fixable Yellow Dead Cell Stain (Thermo Fisher Scientific). For phosphoflow staining, cells were stimulated with 20 ng/ml of recombinant human IL-23 (carrier free, R&D) for 15 mins and were permeabilized by True-Phos™ Perm Buffer (BioLegend) following the manufacturer's protocol. AbC™ Total Antibody Compensation Beads (Cat. No. A10497, Life Technologies) and ArC™ Amine Reactive Compensation Beads (Cat. No. A10346, Life Technologies) were used for compensation. Data were acquired on an LSRFortessa™ flow cytometer (BD Biosciences) using BD FACSDiva™ software in the Cytometry Core at the University of Florida and analyzed by FlowJo software (Version 10, Tree Star Inc.).

### 2.7. Histology

The surgical tissues containing the structures of mucosa, submucosa, and muscular layer were fixed in 10% neutral-buffered formalin (Fisher Scientific) for at least 18 hrs. The fixed tissues were then embedded in paraffin, sectioned, and stained with hematoxylin and eosin.

### 2.8. Gene Expression Heatmap

Total RNA was isolated from intestinal tissues of Crohn's disease patients using QIAzol Lysis Reagent (QIAGEN), and a two-step RT-PCR was performed using the SuperScript® VILO cDNA Synthesis Kit (Thermo Fisher Scientific) for reverse transcription (RT). Quantitative RT-PCR was done using TaqMan® Gene Expression Array Plates (96-well Standard Human Immune Response Array, Cat. No. 4414073, TaqMan) on the QuantStudio 7 Flex Real-Time PCR System (Applied Biosystems). The expression levels of each gene were calculated using the 2^-*ΔΔ*Ct value, and GAPDH was used as endogenous control gene whereas normal unaffected tissue sample was used as reference control. Heatmap of RT-PCR data was generated using R programming.

### 2.9. Statistical Analysis

The correlation of surgery number and *STAT3 rs744166* risk allele was tested by a GENMOD procedure in SAS with the adjustment of age at diagnosis. Statistical analysis was performed using GraphPad Prism 6 software (GraphPad Software, CA), and all comparisons between experimental groups were evaluated by the unpaired Mann-Whitney *t* test. Differences with a value of *p* < 0.05 were considered as significant and were indicated by an asterisk.

## 3. Results

### 3.1. *STAT3 rs744166* Risk Allele “A” Carriage Positively Correlates with Clinical Outcomes of Crohn's Disease Patients

**Figure 1:**
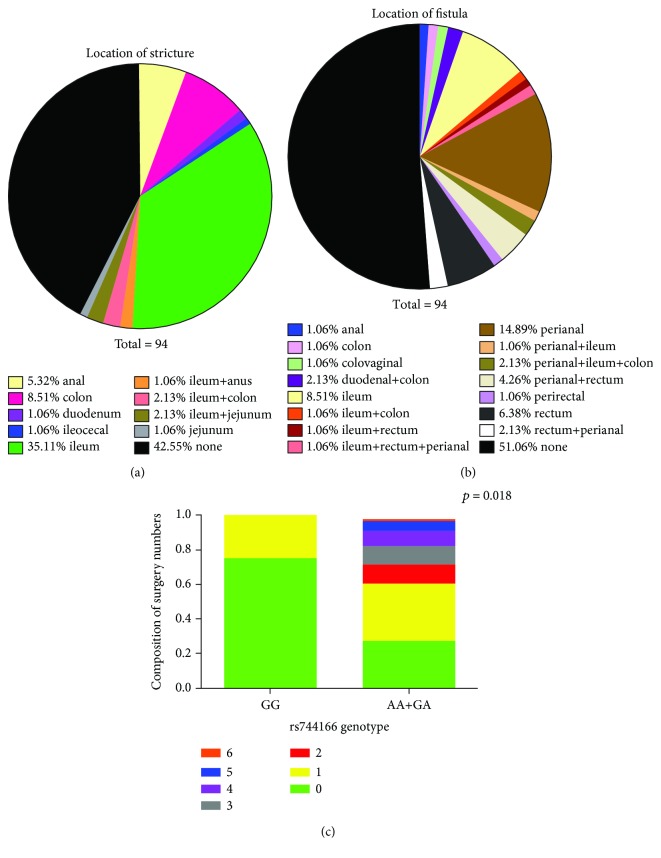
*STAT3 rs744166* risky allele “A” positively correlates with surgery numbers in Crohn's disease patients. Patients in this study suffered stricture and fistula at various sites of the gastrointestinal tract. The locations of strictures was shown in (a), and (b) demonstrated various locations of fistula in this cohort of patients. (c) The correlation of surgery numbers and carriage of risky allele “A” was tested by a GENMOD procedure in SAS with the adjustment of age of diagnosis. Different surgery numbers were represented as different colors.

Surgery is not commonly required for CD patients; however, depending on disease severity and chronic relapse of inflammation, surgical removal of the inflamed tissues is needed in certain cases. Even though the carriage of *STAT3 rs744166* risk “A” allele is relatively high in healthy individuals, which is 56.4% compared to 63.6% carriage in CD patients [26], it has been indicated in several studies that *STAT3 rs744166* is associated with Crohn's disease susceptibility [18, 27]. Herein, we first genotyped our cohort of 94 CD patients and found that 87.2% of this cohort carried *STAT3 rs744166* risk allele “A,” heterozygous or homozygous (Table 1). In order to investigate whether this STAT3 single nucleotide polymorphism (SNP) was associated with clinical outcomes (Supplementary Table 1), we used the number of surgeries that these patients received after diagnosis to represent disease severity (Table 2). We observed that more than 60% of patients do not respond well to drug treatment and eventually need surgery. Moreover, 37.22% of patients have received multiple surgeries (surgery numbers ≥2). In addition, 57.45% of patients have strictures located in various sites, and 48.94% of patients suffered from fistula at different locations (Figures 1(a) and 1(b)). However, there is no correlation between *STAT3 rs744166* and the presence of stricture or fistula. Furthermore, a statistical correlation test of numbers of surgeries and the SNP was performed, and we found that there was a positive correlation between *STAT3 rs744166* risky allele carriage (G/A or A/A) and surgery numbers (Figure 1(c)). Taken together, these results implied a potential involvement of dysfunctional STAT3 activity in the chronic relapse of Crohn's disease patients.

### 3.2. Increased *stat3* and Its Target Gene Expression in the Inflamed Ileum of Crohn's Disease Patients

**Figure 2:**
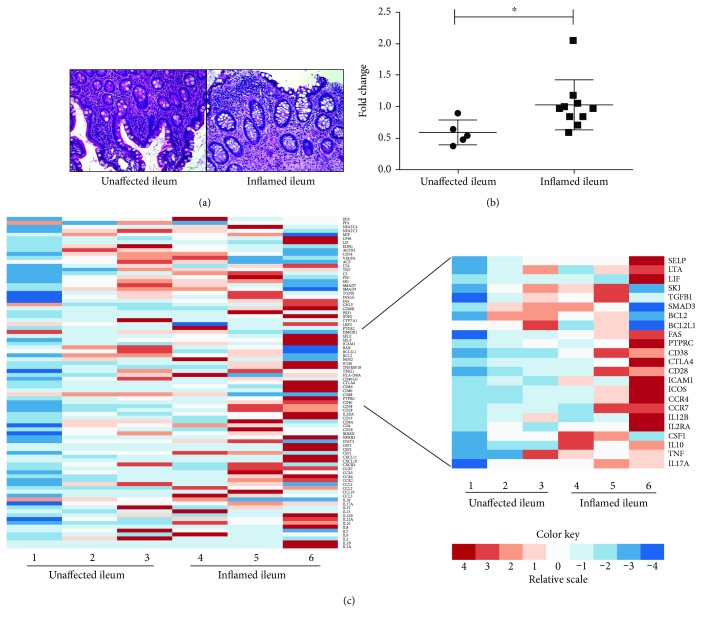
Increased STAT3 target gene expression and *stat3* mRNA expression in the inflamed ileum of Crohn's disease patients. (a) Representative H&E staining illustrates histological appearance in unaffected and inflamed terminal ileum of CD patients. Compared to unaffected ileum, the inflamed ileum showed distinct histological signs of inflammation, including increased infiltration of immune cells in the lamina propria, loss of intact small intestinal architecture, and ulceration. Images were obtained under 20x magnification. Scale bar = 50 *μ*m. (b) *stat3* mRNA expression was upregulated in the inflamed ileum of CD patients (*n* = 10), compared to unaffected ileum tissues (*n* = 5). The gene expression fold change was calculated as 2^-*ΔΔ*Ct value, using GAPDH as endogenous control. Statistical analysis was done by the unpaired Mann-Whitney *t* test, *α* = 0.05. (c) Altered expression pattern of inflammatory genes in the inflamed tissues of CD patients. Among these genes, an increased STAT3-related gene expression was observed in the inflamed ileum of CD patients. GAPDH was used as endogenous control.

To further investigate STAT3 activity in the disease state, we evaluated *stat3* mRNA levels in the inflamed ileum tissues from CD patients who received more than 2 surgeries. Based on distinct histological features, for instance, ulceration, crypt abscesses, lymphoid aggregates, and architectural distortion, we defined surgical resections from CD patients as microscopic unaffected ileum and inflamed ileum (Figure 2(a)). We observed a significantly increased level of *stat3* mRNA expression in the inflamed ileums, compared to unaffected ileum of CD patients (Figure 2(b)), suggesting STAT3 activity was upregulated under inflammation. In addition, the *stat3* expression level was also increased in the peripheral blood of CD patients, compared to healthy donors (Supplementary Figure 1). Furthermore, an altered expression pattern of inflammatory genes was observed in the inflamed ileum of CD patients, compared to unaffected ileum (Figure 2(c), A). Among those inflammatory genes, we further evaluated the STAT3-related genes, such as *Il-17a*, *tnf*, and *Il-10*, and found increased expression levels of those STAT3 target genes in inflamed ileum tissues (Figure 2(c), B). The evaluated gene IDs were listed in Supplementary Table 2, and the calculated expression levels of each gene were reported in Supplementary Table 3. In order to investigate whether *STAT3 rs744166* associates with the increase of STAT3-related gene expression during inflammation, we then genotyped these patients. As the result, the genotype of sample 1, 2, and 5 was A/A, and the genotype of sample 6 was GG. Samples 3 and 4 were the same individual before and after treatment of 5-month Ustekinumab (anti-IL12/23 p40 antibody), and this patient was a heterozygous “A” carrier. However, the data implied this increased proinflammatory gene expression level was not associated with *STAT3 rs744166* risk allele “A” but correlated with the inflammation level.

### 3.3. Increased Activation of STAT3 Signaling in the Inflamed Ileum of Crohn's Disease Patients

**Figure 3:**
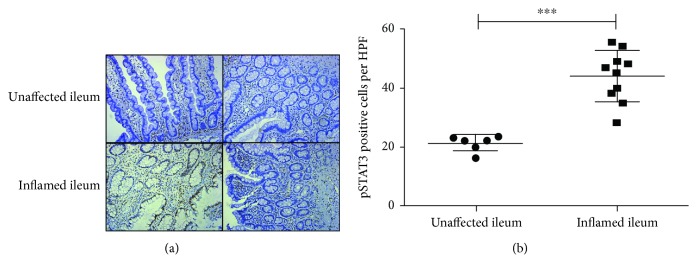
Increased activation of STAT3 signaling in the inflamed ileum of Crohn's disease patients. (a) Representative images of pSTAT3-Y705 IHC staining on unaffected (*n* = 6) and inflamed ileum of CD patients (*n* = 10). pSTAT3^+^ cells were shown as brown dots. Images were obtained under 20x magnification. Scale bar = 50 *μ*m. (b) Statistical analysis of pSTAT3-Y705^+^ cells per HPF in unaffected and inflamed ileum of CD patients. Unpaired Mann-Whitney *t* test was performed, *α* = 0.05.

To examine the activation of STAT3 signaling, we performed IHC staining of pSTAT3-Y705 on paraffin-embedded tissue sections of CD patients. The phosphorylated STAT3 signal was detected mainly in those immune cells located in the lamina propria, as well as intestinal epithelial cells for tissue repair in the inflamed ileum [28] (Figure 3(a)). pSTAT3^+^ cells were counted per high-power field (HPF) in unaffected tissues and inflamed tissues of CD patients, and an increased activation of STAT3 was observed in the inflamed ileum (Figure 3(b)).

### 3.4. Activation of STAT3 Signaling in Innate Lymphoid Cells

**Figure 4:**
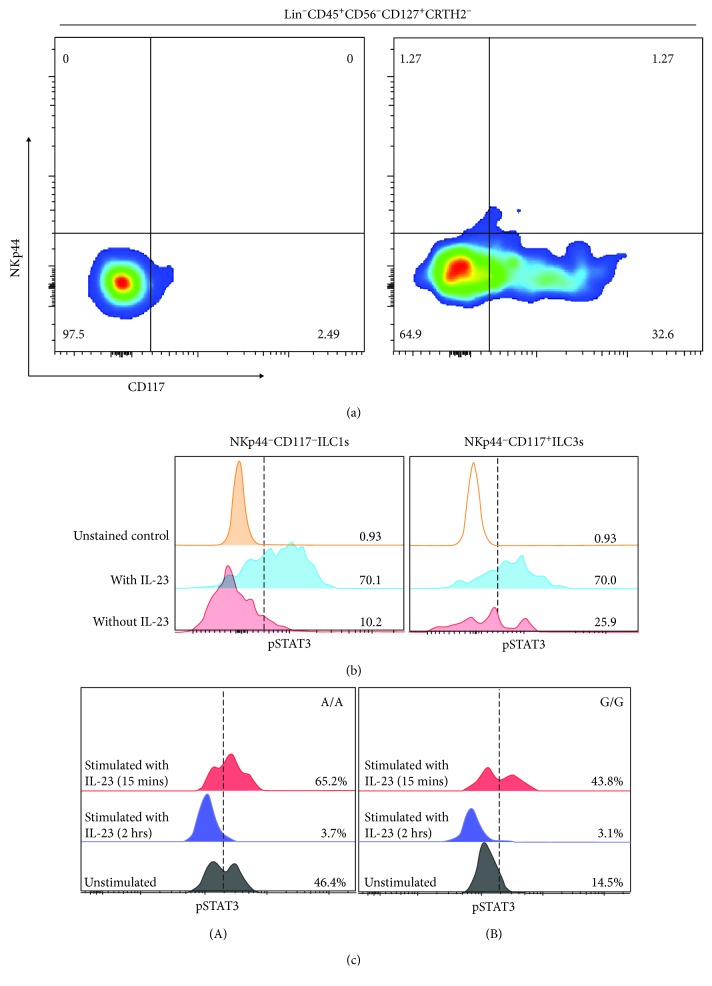
Activation of STAT3 signaling in innate lymphoid cells in response to IL-23 stimulation. (a) PBMCs from healthy donor were stimulated by 20 ng/ml of IL-23 for 15 minutes. After gating Lin^−^CD45^+^CD127^+^CRTH2^−^ cells, the ILC population was further gated based on CD117 and NKp44 expression. The CD117^+^NKp44^−^ ILC3 population (NCR^−^ILC3 phenotype) was induced by IL-23 stimulation. (b) IL-23 activated STAT3 signaling in both the CD117^−^NKp44^−^ ILC1 and CD117^+^NKp44^−^ ILC3 subsets. (c) After gating Lin^−^CD45^+^CD56^+^CD127^+^CRTH2^−^ ILCs, the tyrosine phosphorylation level of STAT3 was evaluated in ILCs. Donor with A/A genotype was shown in (A), and donor with G/G genotype was demonstrated in (B). The pSTAT3 level after 15 mins or 2 hrs stimulation of IL-23 indicated there was no delayed dephosphorylation in donors, suggesting risky allele “A” carriage does not affect the dephosphorylation of STAT3 in ILCs.

STAT3 has been shown that plays a critical role in Th17 development and function, responding to IL-6 and IL-23 signals. As the innate compartment of T helper cells, ILCs also respond to IL-23 stimulation, and the activation of STAT3 was crucial to the IL-22 production of ILC3s [22]. However, it is unknown whether STAT3 involves in ILC1-to-ILC3 transdifferentiation as IL-23 is the driving cytokine for this plasticity [15]. We have observed differential frequency of Lineage^−^CRTH2^−^CD45^+^NKp44^−^CD117^−^CD127^+^ILC subset in the inflamed terminal ileum of CD patients [13], suggesting there was a dysregulated ILC plasticity in CD patients. Herein, we evaluated pSTAT3 levels in ILC subsets in response to IL-23 stimulation using PBMCs from healthy donor, and we also desired to investigate whether the carriage of *STAT3 rs744166* “A” allele correlates with the altered activation level of STAT3 in ILCs. As the result, the NCR^−^ILC3s (Lin^−^CD45^+^CD56^−^CD127^+^CRTH2^−^CD117^+^NKp44^−^ cells) responded rapidly to IL-23 stimulation (Figure 4(a)), and these cells are more progenitor-like cells which are able to differentiate to NCR^+^ILC3s [29]. We then examined the phosphorylation level of STAT3 following IL-23 in each ILC subset (Figure 4(b)). To our surprise, the pSTAT3 signal was also detected in the Lin^−^CD45^+^CD56^+^CD127^+^CRTH2^−^CD117^−^NKp44^−^ ILC1s, which were reported accumulated in the inflamed ileum of CD patients [13]. This result suggested that the activation of STAT3 signaling may be the first step of a signaling cascade for ILC1-to-ILC3 plasticity and implied that STAT3 potentially has a regulatory role for ILC1 functioning. Furthermore, we eager to investigate whether the *STAT3* genotypic variant has impact on cellular STAT3 activation in ILCs in response to IL-23. Here, we used PBMCs from donors with A/A and G/G genotypes to evaluate the potential effect of *STAT3* “A” risk allele carriage on the phosphorylation of STAT3 in ILCs. Notably, the result revealed that donors carrying *STAT3 rs744166* “A” risk allele exhibited a higher basal level of STAT3 tyrosine phosphorylation as well as an increased IL-23-stimulated pSTAT3 level in Lin^−^CD45^+^CD56^+^CD127^+^CRTH2^−^ ILCs (Figure 4(c), A: A/A donor; B: G/G donor). By examination of pSTAT3 after 15 mins and 2 hrs of IL-23 stimulation, we illustrated that there was no delayed dephosphorylation of STAT3 in ILCs. In addition, the carriage of “A” allele did not impact the dephosphorylation of STAT3.

## 4. Discussion

Despite the introduction of numerous biological agents, including anti-TNFs, anti-integrin blockers, and more recently, anti-IL12/23 drugs, for Crohn's disease patients in remission, relapse rates at one, two, five, and ten years are estimated at 20%, 40%, 67%, and 76%, respectively [30]. Therefore, a better understanding of CD pathogenesis is needed in order to select more specific and effective treatment regimens. There are several newly approved small molecule inhibitors targeting JAK-STAT signaling; however, the responsiveness varies by individual [31]. This study was mainly focused on investigating the role of STAT3 in the pathogenesis and progression of Crohn's disease. We positively correlated the prevalence of the *STAT3 rs744166* risky allele “A” with disease severity in terms of surgery numbers (Figure 1). In addition, we observed an increased level of *stat3* mRNA, STAT3-related gene expression, as well as activation of STAT3 signaling (phosphorylated STAT3) in inflamed ileum tissues of CD patients compared to unaffected ileum (Figures 2 and 3). To investigate STAT3 activity in the innate lymphoid cells of particular interest, we then specifically examined pSTAT3 in each of the ILC subsets in response to IL-23 stimulation (Figure 4). Notably, we found that STAT3 signaling was not only activated in the NCR^−^ILC3 subset but also activated in Lin^−^CD45^+^CRTH2^−^CD127^+^CD117^−^NKp44^−^ cells. Moreover, we illustrated the carriage of *STAT3* genotypic variant results in an increased basal and IL-23-stimulated STAT3 tyrosine phosphorylation, but it does not affect the dephosphorylation of STAT3 in ILCs.

Previous studies have focused largely on the role of adaptive immunity and how it drives the pathogenesis as well as progression of Crohn's disease. The research on innate immune responses have been continuously expanded over the past two decades [32]. As the important tissue-resident guardian, innate lymphoid cell has been investigated in both steady state and diseased state, suggesting the important role of ILCs in mucosal immunity [33]. Specifically in Crohn's disease patients, our previously published study showed that the accumulation of IFN-*γ*-producing Lin^−^CD45^+^CRTH2^−^CD127^+^CD117^−^NKp44^−^ cells in the inflamed terminal ileum of CD patients correlates with disease severity [13]. However, the paradox we have observed is an increased STAT3 activity along with decreased ILC3 numbers in the inflamed tissues of CD patients. Bernink et al. not only proposed that IL-23 triggered ILC1-to-ILC3 transdifferentiation but also pointed out that sustained exposure of IL-23 was able to promote the reverse conversion [15]. Other groups also pointed out that IL-23 responsive ILC3s induced pathogenesis of neonatal intestinal inflammation [34]. Additionally, it has been shown that STAT3 phosphorylation in ILC3s was suppressed by the presence of T_reg_ and Th17 cells [35]. In this study, we revealed that the *STAT3* genotypic variant *rs744166* risk allele “A” was associated with increased basal and IL-23 triggered the pSTAT3 level in ILCs, but there was no impact on the dephosphorylation of STAT3 in those genetically susceptible individuals. Taken together, we suggested that investigation of STAT3 signaling in a cell-specific manner is needed for future researches. IL-23-induced activation of STAT3 signaling in CD117^−^NKp44^−^ ILC subset suggested that STAT3 plays a role in the initiation of ILC plasticity and also potentially involves in regulating Lin^−^CD45^+^CRTH2^−^CD127^+^CD117^−^NKp44^−^ ILC1 function. More in depth investigation on exact gene regulation of STAT3 in ILCs is necessary to further explore the exact molecular mechanism of ILC plasticity and heterogeneity property.

## 5. Conclusion

Overall, this study illustrated that increased STAT3 activity was associated with the pathogenesis and progression of Crohn's disease. Particularly, activated STAT3 signaling in IL-23 responsive ILCs may lead to the chronic relapse of CD. Notably, individuals carrying *STAT3 rs744166* “A” risk allele exhibited increased basal and IL-23-stimulated STAT3 tyrosine phosphorylation in the peripheral ILCs. The results provide a novel concept of a potential mechanism of ILC plasticity and also contribute to potential mechanistic strategies for use in personalized medicine in the treatment of Crohn's disease patients.

## Figures and Tables

**Table 1:** *STAT3 rs744166* genotyping in a cohort of Crohn's disease patients.

Genotype	Number	Frequency
GG	12	12.8%
GA	41	43.6%
AA	41	43.6%

**Table 2:** Surgery numbers in risky allele carrier and nonrisky allele carrier among the cohort of Crohn's disease patients.

Genotype		Surgery numbers							Total
		0	1	2	3	4	5	6	
GG	Number	9	3	0	0	0	0	0	12
	Percentage	9.57%	3.19%	0%	0%	0%	0%	0%	12.77%

AA+GA	Number	26	21	11	9	8	6	1	82
	Percentage	27.66%	22.34%	11.70%	9.57%	8.57%	6.38%	1.06%	87.23%

Total	Number	35	24	11	9	8	6	1	94
	Percentage	37.23%	25.53%	11.70%	9.57%	8.51%	6.38%	1.06%	100%

## Data Availability

The genotyping, clinical outcomes of patients, and flow cytometry data used to support the findings of this study are available from the corresponding author upon request.

## Supplementary Materials

Supplementary 1 Supplementary Table 1: clinical characteristics of enrolled Crohn's disease patients.

Supplementary 2 Supplementary Table 2: list of human immune response genes.

Supplementary 3 Supplementary Table 3: quantitative expression levels of human immune response genes.

Supplementary 4 Supplementary Figure 1: RT-PCR of stat3 mRNA level in PBMCs from donor and Crohn's disease patients.

Supplementary 5 Supplementary Figure 2: positive and negative controls of pSTAT3-Y705 IHC staining.

Supplementary 6 Supplementary Figure 3: gating strategy of ILC subsets using healthy donor PBMCs.
