# Supplementary material for: STAT3 Genotypic Variant rs744166 and Increased Tyrosine Phosphorylation of STAT3 in IL-23 Responsive Innate Lymphoid Cells during Pathogenesis of Crohn's Disease
Source: J Immunol Res. 2019 Jun 19;2019:9406146. doi: 10.1155/2019/9406146 (PMC6610725; doi:10.1155/2019/9406146)
Supplement: Supplementary 4 — Supplementary Figure 1: RT-PCR of stat3 mRNA level in PBMCs from donor and Crohn's disease patients. [file 9406146.f4.pdf]

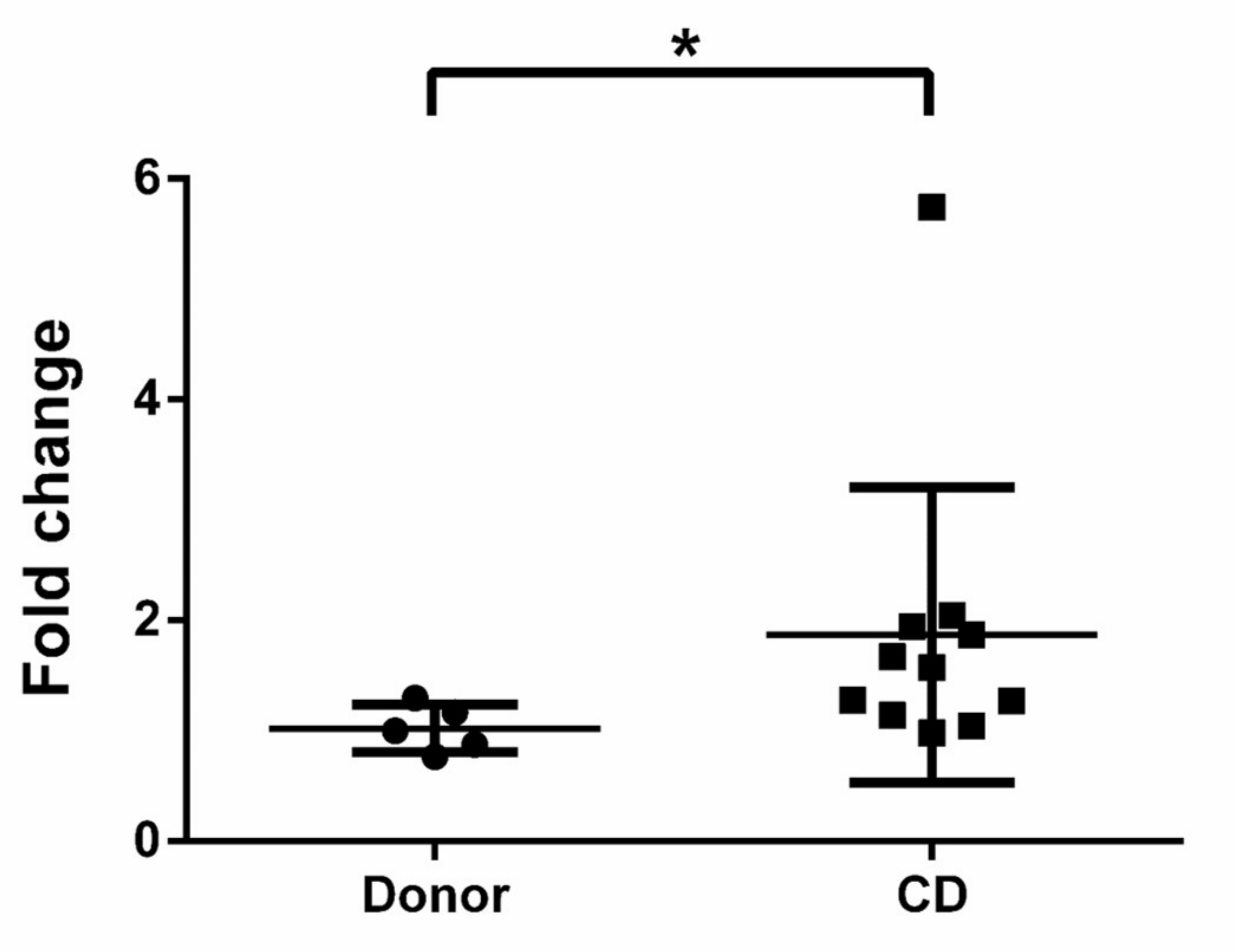

Supplementary Figure 1: Increased level of *stat3* mRNA expression in peripheral blood of Crohn's disease patients (n = 10), compared to healthy donors (n = 5). Unpaired Mann-Whitney *t* test was performed, α = 0.05.
