# Supplementary material for: STAT3 Genotypic Variant rs744166 and Increased Tyrosine Phosphorylation of STAT3 in IL-23 Responsive Innate Lymphoid Cells during Pathogenesis of Crohn's Disease
Source: J Immunol Res. 2019 Jun 19;2019:9406146. doi: 10.1155/2019/9406146 (PMC6610725; doi:10.1155/2019/9406146)
Supplement: Supplementary 5 — Supplementary Figure 2: positive and negative controls of pSTAT3-Y705 IHC staining. [file 9406146.f5.pdf]

Positive control **Rabbit IgG Negative control**

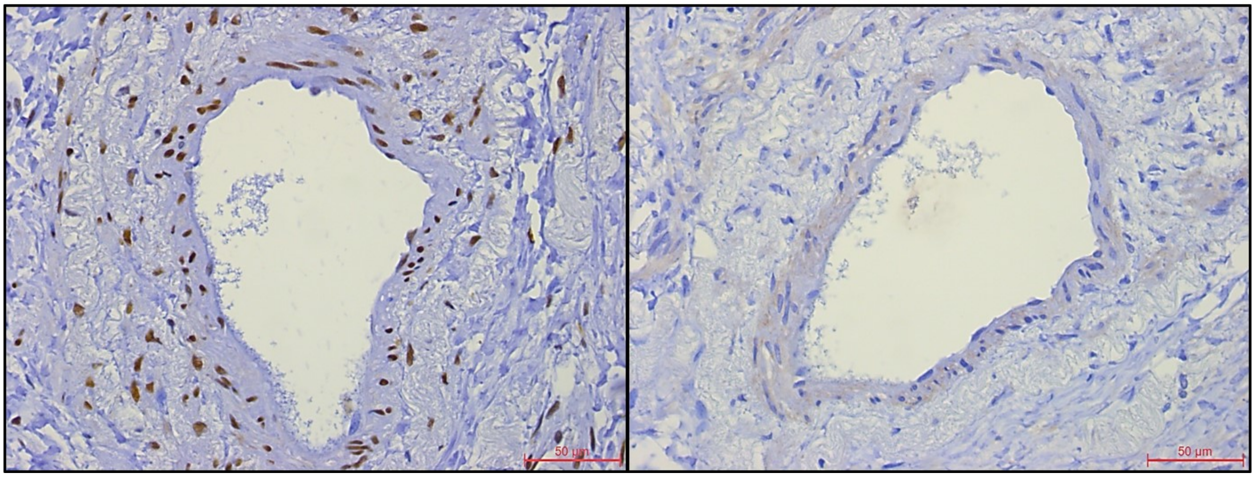

Supplemental figure 2: pSTAT3-Y705 IHC staining positive and negative controls. Images were obtained under 20X magnification. Scale bar = 50µm.
