# Supplementary material for: STAT3 Genotypic Variant rs744166 and Increased Tyrosine Phosphorylation of STAT3 in IL-23 Responsive Innate Lymphoid Cells during Pathogenesis of Crohn's Disease
Source: J Immunol Res. 2019 Jun 19;2019:9406146. doi: 10.1155/2019/9406146 (PMC6610725; doi:10.1155/2019/9406146)
Supplement: Supplementary 6 — Supplementary Figure 3: gating strategy of ILC subsets using healthy donor PBMCs. [file 9406146.f6.pdf]

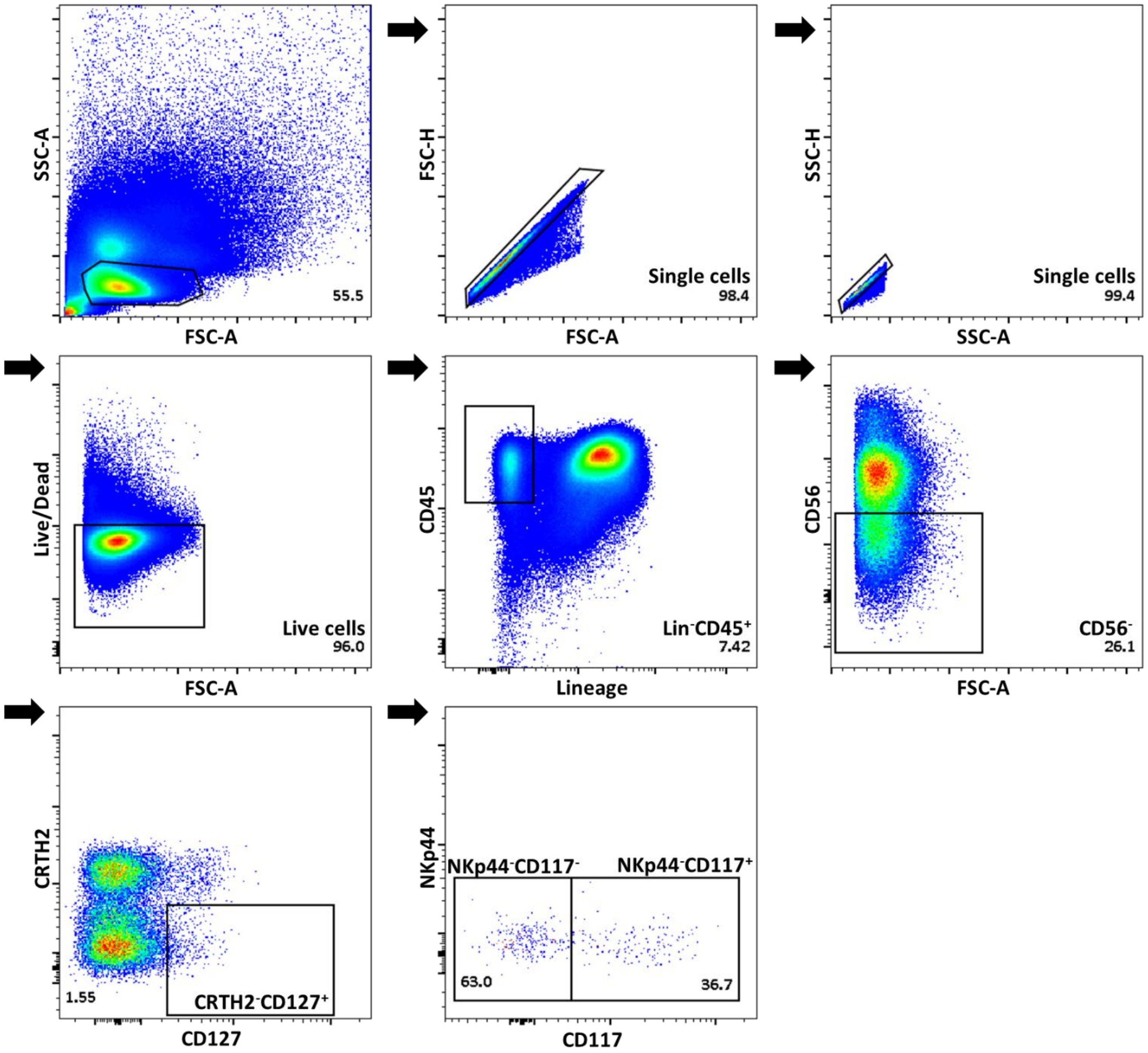

Supplementary Figure 3: Gating strategy of ILC subsets using PBMCs from healthy donors. Isolated PBMCs were first gated by FSC and SSC for single cells, and viability dye was used to eliminate the dead cells. Group 1 and group 3 innate lymphoid cells were then gated as Lineage^{-}CD45^{+}CD56^{-}CRTH2^{-}CD127^{+} cells, and further gated as NKp44^{-}CD117^{-} ILC1s as NKp44^{-}CD117^{+} ILC3s. The compensation was done using compensation beads, and calculation was performed on BD FACSDiva^{TM} Software before running samples.
